# Frequent use of empty *P* *arasa lepida* cocoons by diverse invertebrates

**DOI:** 10.1002/ecy.70592

**Published:** 2026-11-09

**Authors:** Hiroki Akimoto

**Affiliations:** ^1^ Department of Integrative Bioscience and Biomedical Engineering Graduate School of Advanced Science and Engineering, Waseda University Tokyo Japan

**Keywords:** biogenic structures, cocoon reuse, empty cocoons, invertebrate assemblage, Japan, land snails, Limacodidae, *Parasa lepida*

Animal‐built structures can persist in the absence of their builders and continue to shape ecological interactions (Jones et al., 1994). Insect‐built structures can likewise provide habitat for other organisms (Cornelissen et al., 2016). Among these structures, abandoned nests and empty cocoons can serve as shelters or nesting sites for secondary occupants (Bogusch et al., 2025; Coppel, 1960; Furusawa & Okamiya, 2022; Raath et al., 2017). Cocoons of limacodid moths are of particular interest in this context because the family is distributed worldwide (Epstein et al., 2025). The larvae typically construct hard silk cocoons incorporating calcium oxalate (Epstein & Fiesler, 2023). They feed on trees and shrubs (Lill et al., 2006), and some species can become sufficiently abundant to cause substantial canopy defoliation (Duke, 2002). In *Parasa lepida*, cocoons can persist on tree trunks for several years (Yamazaki et al., 2007) and occur at high local densities (Sawada et al., 2008), potentially providing numerous microhabitats for secondary occupants. Nevertheless, secondary occupancy of limacodid cocoons has received little systematic attention.

On 21 January 2026, live land snails were found lodged in the emergence hole of an empty *P. lepida* cocoon on Hachijojima Island, Japan (Figure 1a). This observation prompted a survey of empty cocoons to quantify secondary occupancy and document occupant taxa and life stages.

**FIGURE 1:**
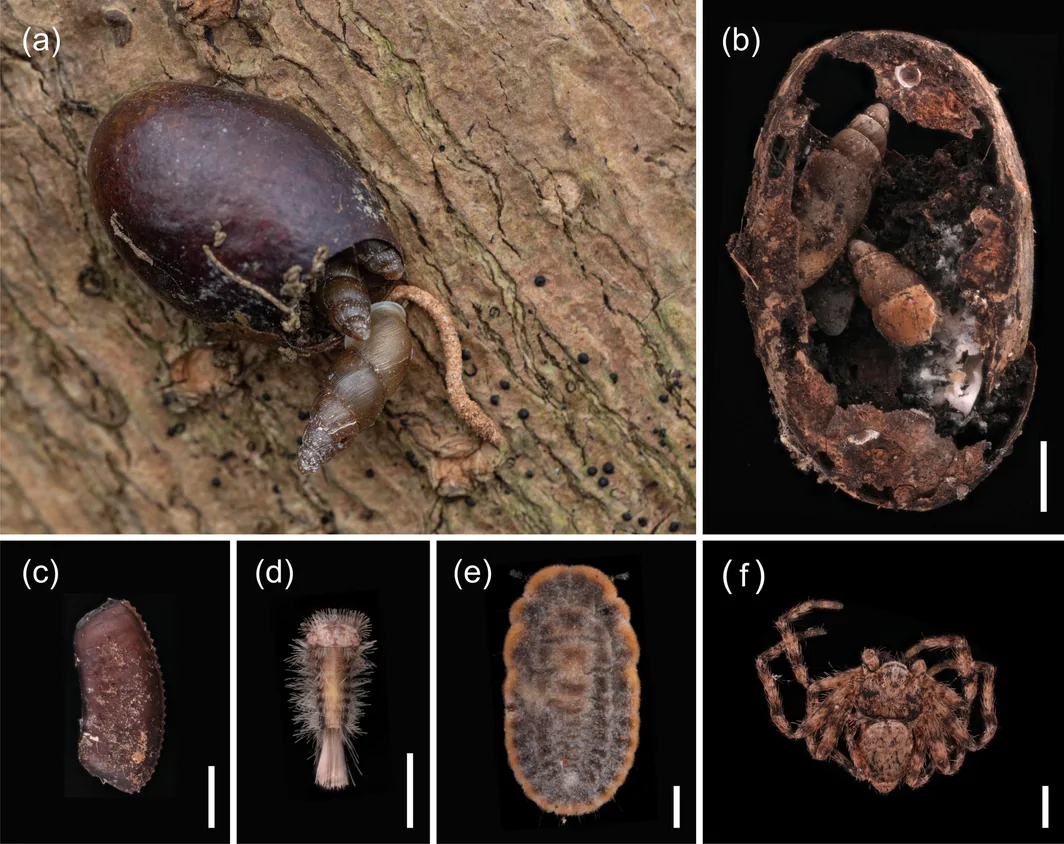
Examples of diverse secondary occupants recorded in empty cocoons of *Parasa lepida* (Limacodidae) on Hachijojima, Japan, 21–22 January 2026. (a) Land snails (Clausiliidae) and a bagworm (Psychidae) protruding from the emergence hole of a bark‐attached cocoon. (b) Interior of an opened cocoon containing clausiliid snails and katydid eggs (*Holochlora* sp., Tettigoniidae). (c) A cockroach ootheca (*Megamareta pallidiola*, Pseudophyllodromiidae). (d) A fringed millipede (*Eudigraphis* sp., Polyxenidae). (e) A scale insect (*Drosicha corpulenta*, Monophlebidae). (f) A running crab spider (*Philodromus spinitarsis*, Philodromidae). Scale bars in panels (b–f): 2 mm. Photographs by Hiroki Akimoto.

Between 21 and 22 January 2026, 19 trees were sampled at multiple sites on Hachijojima (33.08°–33.15° N, 139.74°–139.79° E; elevation 25–171 m). Trees were opportunistically selected if they bore at least five cocoons with apparent moth‐emergence holes within 2 m of the ground. Cocoons were excluded from sampling if only the silk attachment pad remained or if the wall was too damaged for an intact chamber to be recognized. To standardize sampling effort among trees, five qualifying cocoons were collected from each selected tree without preference for those with externally visible occupants (*n* = 95). Each collected cocoon was treated as a sampling unit, and the presence or absence of secondary occupants was recorded. The 19 sampled trees represented seven species. Tree IDs, tree species, and numbers of collected, occupied, and unoccupied cocoons per tree are provided in Appendix S1: Table S1.

Ants and fringed millipedes that exited the cocoons during field handling were preserved in 70% ethanol. The cocoons were opened and their contents examined under a stereomicroscope. The outer length and width of each cocoon were measured with digital calipers. The cocoons were identified as *P. lepida* cocoons based on characteristic external morphology. A cocoon was regarded as empty if it had a circular moth‐emergence hole and dissection confirmed that no living limacodid larva or pupa remained inside. Secondary occupancy was recorded if one or more invertebrates either exited the cocoon during field handling or were found inside its silk chamber during dissection; organisms resting only on the outer surface were not counted. Occupancy therefore indicated presence at the time of collection but not the duration of cocoon use.

Secondary occupants were identified morphologically to the lowest possible taxonomic rank. Within a cocoon, each morphologically distinguishable taxonomic unit was counted once as a morphotaxon, yielding morphotaxon richness rather than individual abundance.

To account for nonindependence among cocoons sampled from the same tree, occupancy was modeled using an intercept‐only binomial generalized linear mixed model (GLMM) with a logit link and tree identity as a random intercept. The model was fitted in R version 4.5.2 (R Core Team, 2025) using the glmer function in lme4 (Bates et al., 2015).

Of the 95 empty cocoons (12–19 × 8–13 mm), 65 contained at least one secondary occupant (68.4%). The binomial GLMM accounting for tree identity yielded a conditional occupancy estimate of 69.3% (95% confidence interval: 58.1–80.8%). Among occupied cocoons, mean morphotaxon richness was 1.72 per cocoon (SE = 0.11, range = 1–5, *n* = 65). Land snails were the most frequent secondary occupants, occurring in 22 cocoons (23.2%), with one to six individuals per cocoon. Fringed millipedes occurred in 17 cocoons (17.9%), spiders in 16 (16.8%), scale insects in 12 (12.6%), and hymenopterans in 11 (11.6%), including two ant species in 10 cocoons (10.5%) (Figure 2). Because colony status was not confirmed, groups of adult ants were treated as aggregations rather than colonies; the two largest, in separate cocoons, contained 308 and 67 adults. The most frequent pairwise co‐occurrences were fringed millipedes with spiders and katydid eggs with land snails, each recorded in four cocoons. The records spanned multiple life stages, including eggs (Araneae, Orthoptera, Phasmatodea, and unidentified insects), oothecae (Blattodea), and larvae or juveniles (e.g., Psychidae, Pyralidae, and Tineidae) (Figures 1 and 2). On each tree, one to five of the five sampled cocoons were occupied, with occupancy recorded on all 19 trees (Appendix S1: Table S1).

**FIGURE 2:**
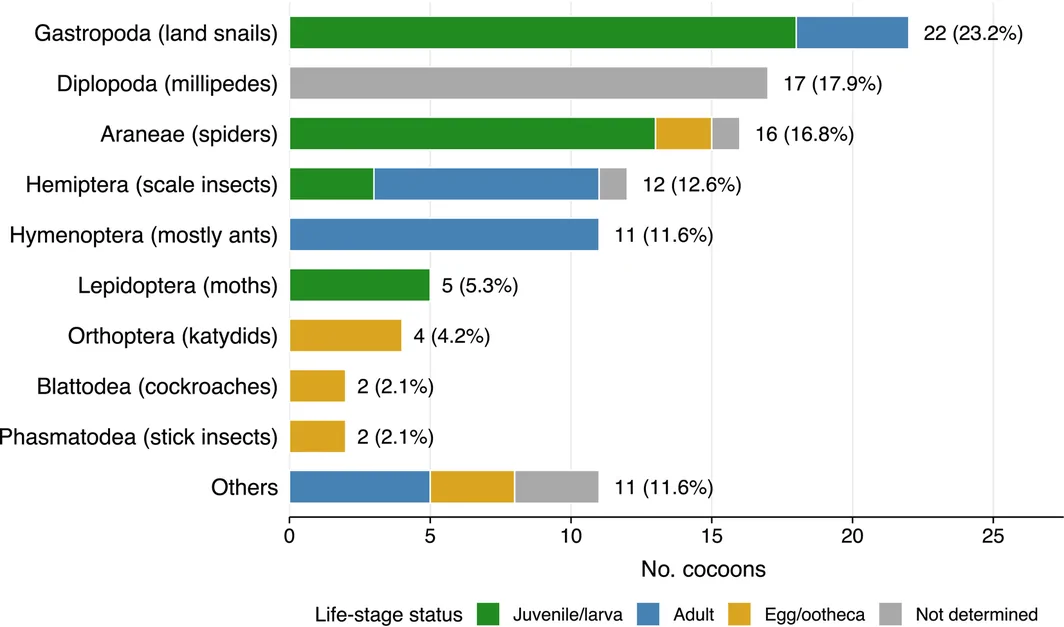
Taxonomic composition and life‐stage status of secondary occupants recorded inside empty *Parasa lepida* cocoons on Hachijojima, Japan, 21–22 January 2026. Of the 95 cocoons collected from 19 cocoon‐bearing trees, 65 contained at least one secondary occupant. Stacked horizontal bars show the number of occupied cocoons in which each taxonomic category was recorded, with colors indicating life‐stage status (juvenile/larva, adult, egg/ootheca, or not determined). Labels show counts and percentages relative to all 95 sampled cocoons. A single cocoon could be counted in more than one taxonomic category. Eggs and oothecae are grouped in the same life‐stage category in the figure. “Others” comprises Coleoptera (two cocoons), Collembola (three), Diptera (three), and unidentified egg deposits (three cocoons). For plotting purposes, when multiple life stages of the same taxonomic category occurred in a single cocoon, that cocoon was counted once for that category and assigned to juvenile/larva, egg/ootheca, adult, or not determined, in that order.

Empty *P. lepida* cocoons were small, discrete arboreal chambers occupied by diverse invertebrates. By scoring individual cocoons for occupancy and documenting their occupants and associated life stages, this study adds a quantitative cocoon‐level perspective to previous reports of secondary use of empty cocoons and abandoned nests. Because *P. lepida* cocoons can persist on tree trunks for several years (Yamazaki et al., 2007), they may remain available to other organisms after their original use by the moth has ended. Eggs, oothecae, and larvae or juveniles were also recorded, raising the possibility that cocoons were used for reproduction or development, although these functions were not directly tested. Because only trees bearing at least five qualifying cocoons within 2 m of the ground were sampled, patterns on trees with fewer cocoons or in higher canopy strata remain unknown. Within this sampling frame, however, the frequency and variety of these records suggest a broader pattern of arboreal microhabitat use rather than isolated instances of shelter use.

The high occupancy observed here provides a rationale for comparative surveys across limacodid taxa. Because only one species was examined, however, the present data cannot determine whether secondary occupancy varies among species or with cocoon traits. Cocoons of two limacodid species differed in hardness and conspicuousness (Furukawa et al., 2021), and ant occurrence was associated with cocoon hole type in another moth‐cocoon system (Raath et al., 2017). Comparative surveys could test whether cocoon hardness and conspicuousness predict occupancy frequency and occupant composition, and whether emergence‐opening size and accessibility are associated with occupant identity, body‐size distribution, and life‐stage structure.

## CONFLICT OF INTEREST STATEMENT

The author declares no conflicts of interest.

## Supporting information


Appendix S1.

## Data Availability

Data and R code (Akimoto, 2026) are available in Dryad at https://doi.org/10.5061/dryad.8sf7m0d4b.
