# Supplementary material for: Frequent use of empty P arasa lepida cocoons by diverse invertebrates
Source: Ecology. 2026 Nov 9;107(11):e70592. doi: 10.1002/ecy.70592 (PMC13748790; doi:10.1002/ecy.70592)
Supplement: Supplementary file 1 [file ECY-107-e70592-s001.pdf]

Appendix S1

Frequent use of empty *Parasa lepida* cocoons by diverse invertebrates

Hiroki Akimoto

Ecology

Table S1. Tree-level summary of empty *Parasa lepida* cocoons collected on Hachijojima, Japan, 21–22 January 2026. Five cocoons were collected from each of 19 trees (n = 95). A cocoon was scored as occupied when one or more invertebrates either exited it during field handling or were found inside the silk chamber during dissection; organisms resting only on the outer surface were not counted.

Tree ID	Tree species	Cocoons collected	Cocoons occupied	Cocoons unoccupied
T01	*Cinnamomum yabunikkei*	5	5	0
T02	*Machilus thunbergii*	5	4	1
T03	*Machilus thunbergii*	5	4	1
T04	*Prunus × yedoensis*	5	1	4
T05	*Cinnamomum yabunikkei*	5	2	3
T06	*Eurya japonica*	5	2	3
T07	*Eurya japonica*	5	4	1
T08	*Cinnamomum yabunikkei*	5	3	2
T09	*Cinnamomum yabunikkei*	5	5	0
T10	*Machilus thunbergii*	5	4	1
T11	*Machilus thunbergii*	5	4	1
T12	*Pittosporum tobira*	5	3	2
T13	*Machilus thunbergii*	5	2	3
T14	*Euonymus japonicus*	5	5	0
T15	*Mallotus japonicus*	5	5	0
T16	*Machilus thunbergii*	5	3	2
T17	*Machilus thunbergii*	5	3	2
T18	*Machilus thunbergii*	5	3	2
T19	*Euonymus japonicus*	5	3	2
**Total**		**95**	**65**	**30**
